# Peripheral Arterial Disease and Ankle-Brachial Index Abnormalites in Young and Middle-Aged HIV-Positive Patients in Lower Silesia, Poland

**DOI:** 10.1371/journal.pone.0113857

**Published:** 2014-12-12

**Authors:** Wiesława Kwiatkowska, Brygida Knysz, Katarzyna Arczyńska, Justyna Drelichowska, Marcin Czarnecki, Jacek Gąsiorowski, Maciej Karczewski, Wojciech Witkiewicz

**Affiliations:** 1 Wrovasc – Integrated Cardiovascular Centre, Regional Specialist Hospital, Research and Development Center in Wroclaw, Wroclaw, Poland; 2 Department of Angiology, Regional Specialist Hospital in Wroclaw, Research and Development Center in Wroclaw, Wroclaw, Poland; 3 Department of Infectious Diseases, Wroclaw Medical University, Wroclaw, Poland; FIOCRUZ, Brazil

## Abstract

**Background:**

Peripheral arterial disease (PAD) is a clinical manifestation of atherosclerosis and mainly refers to elderly patients, having a negative impact on their functionality and quality of life. The findings of previous studies in HIV-infected patients have shown that cardiovascular risk is higher and PAD occurs more frequently than in the general population. There are also contradictory observations. Much less is known about the ankle-brachial index (ABI) value in asymptomatic HIV-infected patients. The aim of this study was to evaluate the prevalence of PAD and ankle-brachial index abnormalities as well as to determine risk factors related to the disease in a group of Polish HIV–positive patients.

**Methods and Findings:**

One hundred and eleven young to middle aged HIV–positive subjects and 40 noninfected subjects were enrolled into the study. Resting ABI measurements were performed and cardiovascular risk was analysed as well. Subgroups were created according to the ABI values: low (PAD), borderline, normal, high and altered ABI. Symptomatic PAD was observed in 2 HIV–positive patients, asymptomatic PAD was not diagnosed. The ABI value is lower and more varied, in 22.5% of the study group altered ABI values were found. Six subjects demonstrated borderline ABI, and 15 high ABI, including >1.4. In the control group no low or very high values were reported. A relation between low ABI and cardiovascular family history and between altered ABI and high–density–lipoprotein cholesterol (HDL–C) level was demonstrated.

**Conclusions:**

In young and middle–aged HIV–positive patients, symptomatic PAD prevalence is comparable to that observed in the overall population. Among asymptomatic patients PAD is not reported. The ABI value in HIV–positive patients is more varied compared to the HIV–negative subjects; the altered ABI shows a strong relation with low HDL–C levels and metabolic syndrome.

## Background

Due to constant development of antiretroviral therapies and significantly prolonged survival in the population of HIV–positive patients, a higher risk for cardiovascular diseases and increased atherosclerosis progression are observed together with infection-related comorbidities [1]. The myocardial infarction risk in HIV-positive patients is over 2 times higher than in the noninfected population. In addition to enhanced coronary atherosclerosis, more advanced atherosclerotic lesions in the cerebral and peripheral arteries are observed [1]. Peripheral arterial disease (PAD) is a frequent clinical manifestation of atherosclerosis in the overall population and mainly refers to elderly patients, having a negative impact on their functionality and quality of life [2], [3]. The disease is often accompanied by atherosclerotic lesions in other vascular areas and the diagnosis of symptomatic or asymptomatic PAD is associated with increased cardiovascular morbidity and mortality as well as all-cause mortality [4], [5]. The diagnostic method for PAD is ankle-brachial index (ABI). PAD is recognised in patients with an ABI value lower than or equal to 0.9 [6]. The borderline ABI value 0.91–0.99 is considered prognostically significant and is associated with higher cardiovascular mortality. High ABI >1.3 (above 1.4 in particular) is considered as a sign of rigidity and noncompressibility of lower limb arteries. Abnormal ABI value in asymtomatic patients is associated with a higher incidence of cardiovascular disease. The most typical symptom of PAD is intermittent claudication. Advanced disease is characterised by rest pain and necrotic lesions of the lower limbs. The findings of previous studies on peripheral arterial disease in HIV-positive patients are inconsistent. Much less is known about the ankle-brachial index value in asymptomatic HIV-infected patients. This is the first report concerning the subject in Polish HIV infected patients. Taking under consideration the higher cardiovascular risk and increased atherosclerosis progression in HIV patients our study was conducted to increase awarness and to broaden the knowledge for early PAD detection and treatment.

The aim of this study was to evaluate the prevalence of PAD and ankle-brachial index abnormalities as well as to determine relation between the ankle-brachial index outcome and cardiovascular or potential risk factors in a group of Polish HIV-positive patients.

## Materials and Methods

### Ethics considerations

All the performed tests except blood sampling during the study were noninvasive. Study protocol was assessed by the ethics committee and the authors of the project obtained the local Bioethic Committee's approval for the study to be conducted. All participants provided their written informed consent to participate in the study, according to the Helsinki Declaration.

### Study area

The study was conducted at the area of Lower Silesia, located in the south-western region of Poland. It is one of the most urbanized and the richest regions of Poland. At the time of the study the region's population counted 2.9 milion inhabitants, which represented 7.54% of the Polish population. The median age of the population in the region was 38.9 years [7].

### Study design and duration

It was an observational, cross-sectional study with participation of two groups: the study group of HIV- infected patients and control group of uninfected, healthy people – residents of the same region. The study was conducted for 10 months since May 2011 to February 2012.

### Study population

The study was conducted in two groups:

#### Study group

The consecutive, routinely scheduled HIV-positive patients attending the Acquired Immunodeficiency Syndrome Outpatient Clinic who fulfilled the inclusion and exclusion criteria were invited to participate in the study. One hundred twenty one patients agreed to participate, of whom 5, based on exclusion criteria were not included (4 patients were excluded because high transaminase level, 1 patient came for a study visit with symptoms of acute illness) and 5 persons missed the study visit. One hundred eleven patients (average age 41.1±9.4) were included in the study. The HIV infection characteristics of the patients living in the Lower Silesia region in which our study was conducted are similar (in the course, route of transmission, co-infections, prevention and treatment) and representative of the entire country. The reported number of HIV-infected people in the region is about 2000. In this out-patient department 1115 patients have been registered. During the study period 750 patients had appointments to attend the clinic. In Poland since 1985 (HIV testing introduction) till the 31^th^ of December 2012 HIV infection was reported in 16314 of Polish citizens and of residents of another citizenship. Among them AIDS (acquired immune deficiency syndrome) was diagnosed in 2848 individuals and 1185 died [8].

#### Control group

The control group included HIV-negative age- and gender-matched healthy subjects recruited contemporaneously (average age 42.3±11.1, proportion of men: 65%) from the study group region, not meeting the project's clinical and laboratory exclusion criteria. These healthy subjects were recruited at random through two general practices in the region's capital and in the province. Seventy one subjects agreed to participate, of whom 31 were not included - two because of positive HBs antigen, 18 because of data in the medical history indicating the occurence of cardiovascular disease or significant chronic disorders and 11 persons missed the study visit). This control group was created to compare the ABI findings between the groups.

The planned study group size was 120 patients, with a control group of 40 healthy persons. The actual study group included 111 patients and the control group 40 individuals.

### Inclusion and exclusion criteria

The inclusion criterion for the study group was diagnosed HIV infection in patients aged 20–60 years. The exclusion criteria were as follows: a currently diagnosed acute medical condition, currently diagnosed AIDS, creatinine levels of above 2 mg%, and over 5 times higher aminotransferase levels (upper limit 40 IU/L). HIV–positive subjects started the study with a known immunological and virological status with regard to both HIV infection and hepatitis B and/or hepatitis C virus co-infection.

The inclusion criterion for the control group was a HIV–negative result in generally healthy patients aged 20–60 years. Cardiovascular disease, significant chronic disorders and all exclusion criteria as for the study group excluded candidates from the control group. The characteristics of HIV infection status of the study group are presented in Table 1.

**Table 1:** Characteristics of HIV-infection status of the study group.

Variable	
Time since diagnosis of HIV infection, years	9 (5–14)
Transmission route	
Heterosexual	31 (27.9)
MSM/bisexual	30 (27.0)
Substance abuse (intravenously)	50 (45.1)
History of AIDS	27 (24.3)
Hepatitis C co- infection	52 (46.9)
Hepatitis B co-infection	22 (19.8)
Patients treated with ARV	98 (88.2)
ART duration, years	6 (4–9.9)
HIV RNA below detection limit	83 (74.8%)
Detectable (40–334000 copies/ml)	28 (25.2%)
CD4+T lymphocyte count cells/µl	546 (422–763)
Nadir CD4+T lymphocyte count cells/µl	229.5 (91–328)
HIV RNA zenith (n = 83), copies/ml	40119 (10113–178250)

Data shown as median and interquartile range or prevalence (number, percent).

MSM – men who have sex with men

ART – antiretroviral treatment

Among the major cardiovascular risk factors, tobacco smoking, hypertension and family history of cardiovascular diseases were the most representative in the study group. The assessment of body posture revealed a significantly lower body mass index in HIV–positive participants compared to the control group. All cholesterol fraction levels were significantly reduced, triglyceride and homocystein levels were higher in the study group. Despite the differences, the general cardiovascular risk was similarly low in both groups (Table 2).

**Table 2:** Comparison of cardiovascular risk factors in the study and control group.

Variable	Study group n = 111	Control group n = 40	P
Age, years	42 (34–47)	46 (31–51)	0.33
Gender (M)	71 (64.0)	26 (65.0)	0.94
Current and past smoking	86 (77.5)	17 (43.0)	**0.0001**
Hypertension	47 (42.3)	10 (25.0)	**0.031**
Diabetes mellitus	3 (2.7)	1 (2.5)	0.69
Lipid disorders	77 (69.4)	22 (55.0)	0.12
Family history	37 (33.3)	6 (15.0)	**0.046**
Obesity	4 (3.6)	3 (7.5)	0.38
Metabolic syndrome according to IDF	22 (19.8)	4 (10)	0.22
Cardiovascular disease	4 (3.6)	0 (0)	0.57
BPs*, mmHg	120 (110–138.75)	120 (113–130)	0.52
BPd**, mmHg	80 (70–90)	80 (70–90)	0.96
TC, mg/dl	190 (158–219.75)	208 (183–236)	0.096
LDL-C, mg/dl	104 (80–128)	128 (97–152)	**0.019**
HDL-C, mg/dl	50 (40.25–60.75)	58.5 (48–70.5)	**0.037**
Non-HDL-C, mg/dl	136 (100–164.75)	149 (125–175)	**0.044**
TG, mg/dl	129 (93–187.75)	101 (69–133)	**0.008**
Lp(a), g/l	0.05 (0.029–0.163)	0.06 (0.02–0.18)	0.49
Pack–years	17 (8.25–26.5)	13.8 (3.3– 29.8)	0.36
CRP, mg/l	0.52 (0.198–1.865)	0.74 (0.18–1.53)	0.91
Fasting glucose, mg/dl	90.8 (85–97)	94.5 (88–101)	0.047
BMI	23.0 (21.01–25.1)	25.7 (24.3–27.3)	**0.0001**
WHR	0.90 (0.84–0.95)	0.89 (0.78–0.94)	0.1
Homocystein, µmol/l	12.6 (10.5–15.758)	11.3 (10.1–13.1)	**0.026**
Fibrinogen, g/l	2.6 (2.2–3.0)	2.9 (2.65–3.3)	**0.0001**
D-dimers, ng/ml	217 (167–341)	246 (171–352)	0.77
Platelets, K/µl	222 (177–277)	260 (217–308)	**0.0025**
cIMT, mm	0.648 (0.597–0.791)	0.541 (0.459–0.621)	**0.0001**
Cardiovascular risk NCEP ATP III, %	2 (1–6.75)	2 (0.99–5.5)	0.45

Data shown as median and interquartile range or prevalence (number, percent).

*BPd – diastolic blood pressure

**BPs – systolic blood pressure

### Methods used for assessment of the relevant parameters

The patients underwent the following vascular assessments: medical history, including medical records and questions about cardiovascular risk factors and events, intermittent claudication and vascular reconstructive procedures. With regard to HIV infection-related data, the following were assessed: duration of infection, transmission route, a history of AIDS, hepatitis B (based on serological examination and presence of HBs antigen) or hepatitis C (based on HCV RNA presence) co-infections, treatment duration and applied antiretroviral pharmacotherapy, current CD4 and nadir CD4 cell counts as well as HIV RNA and HIV RNA zenith titres. Historical data such as HIV infection duration, CD4 T cell count nadir or history of AIDS were abstracted from the available patients health reports.

The pulse rates of peripheral arteries in the lower limbs and murmurs over large arteries were assessed. In the absence of a pulse, additional arterial ultrasound scans were performed. The following parameters were also obtained: the body mass index (BMI) and the waist-hip ratio (WHR). Blood pressure measurements were performed according to the standard practice. Hypertension was diagnosed if the patient had a history of diagnosed and pharmacologically treated hypertension or based on two pressure measurement results equal to or above 140/90 (at least grade one according to WHO). A family history of cardiovascular diseases was determined based on the National Cholesterol Education Program Adult Treatment Panel III (NCEP ATP III) criteria [9]. Metabolic syndrome was recognized according to the International Diabetes Federation (IDF) criteria with typical European waist circumference values [10]. Carotid intima-media thickness (cIMT) was measured – the cIMT measurement methods were presented in the previous paper [11]. Lipid disorders were assessed based on the NCEP ATP III guidelines; borderline levels of total cholesterol and triglycerides were considered abnormal [9]. The cigarette pack-years value was calculated by multiplying the number of packs of cigarettes smoked per day by the number of years the subject has smoked. After a 14-hour fast, blood samples were taken for the laboratory tests, which were performed using standard laboratory methods: hematology analysis, transaminase level (upper limit – 40 IU/l), creatinine, glucose, total cholesterol (TC), HDL cholesterol (HDL-C), triglycerides (TG), lipoprotein(a) Lp(a) (immunonephelometric method), homocysteine, fibrinogen, D-dimers, wide range C- reactive protein (CRP) (latex-enhanced immunoturbidimetry). LDL (LDL-C) cholesterol was calculated according to the Friedewald equation, and non-HDL cholesterol (non-HDL-C) by the formula TC - HDL-C. The level of anti-HIV, anti-HBc and anti-HCV antibodies was determined in the control group using the enzyme immunoassay method with microparticles. Plasma HIV RNA titres were determined using the Roche Amplicor 1.0 standard assay and for CD4 T cell assessment the FACScan (Becton-Dickinson) flow cytometry method was applied.

### ABI measurement

The ABI was assessed using a hand-held continuous wave Doppler device (Doppler SmartDop 45) with an 8 MHz probe. The test was performed in a properly heated room after 5 minutes of rest in the supine position. A pressure cuff was placed on both arms then bilaterally above the ankles. The probe was placed at the cubital fossa behind the medial malleolus and on the dorsal foot for pulse detection. After the cuff was inflated to 20 mm above the audible pulse signal, it was gradually emptied and the pressure value was recorded when the first pulse signal was detected. The systolic blood pressure was determined in both arms, and the ankle systolic blood pressure was determined for the right and left posterior tibial and dorsalis pedis arteries. The ABI was determined by using the higher of the two values from either the posterior or dorsalis pedis artery for each leg and the higher of the two brachial readings. The qualitative statistical analysis of ABI was conducted by choosing the higher or lower abnormal values per patient if both values were abnormal: high or low; if the ABI value was normal in both legs then the lower value was chosen. If a patient had an abnormal value in one leg then we chose this value. Additionally, ABI values measured for the left and right legs of the study group were compared against a control group as quantitative variable.

Symptomatic PAD was diagnosed in patients with intermittent claudication and an ABI value lower than or equal to 0.9. Intermittent claudication was diagnosed based on the Trans-Atlantic Inter-Society Consensus II (TASC II) definition – muscle discomfort in the lower limb, which is reproducibly produced by exercise and relieved by rest [6]. According to TASC II recommendations, all conditions that may cause unusual intermittent claudication or asymptomatic PAD were considered [6]. The diagnostic criterion for asymptomatic PAD was an ABI value lower than or equal to 0.9 with no clinical symptoms.

The ABI was categorised by its values; based on the standard ABI categories, the following patient s were distinguished:

1. A subgroup with low ABI ≤0.9, indicating PAD, n = 2.
2. A subgroup with borderline ABI 0.91–0.99, n = 6.
3. A subgroup with ABI <1.0– combined subgroups 1+2, n = 8.
4. A subgroup with normal ABI 1.0–1.3, n = 88.
5. A subgroup with high ABI >1.3, n = 15.
6. A subgroup with ABI >1.4, distinguished from subgroup 5, n = 5.

Also, a group with altered ABI (subgroups 1+2+5) was created and statistically compared to subgroup 4 (normal ABI). Due to the insufficient sizes of subgroups 1, 2 and 6, they were not subjected to a comparative statistical analysis with the other subgroups (**LINK 1**). The differences in occurrence of cardiovascular risk factors between subgroups 3, 4 and 5 were compared. The ABI findings in the asymptomatic HIV-positive group were compared to those obtained in the control group.

### Statistical analyses

The quantitative variables were presented as the median with the interquartile range. The quantitative differences between the study and control groups as well as between the study subgroups were analyzed, using the Mann-Whitney or Kruskall-Wallis test, as appropriate.

The qualitative variables were presented as the sizes and percentages of specific groups; the qualitative differences were evaluated using the chi-square test or Fisher's exact test. Logistic regression was used to identify risk factors associated with altered ABI. The analysis was performed by means of the R statistical package and MedCalc. Results with a significance level of p <0.05 were found significant.

## Results

### In 22.5% of the cohort subjects altered (low, borderline or high) ABI values were found

Symptomatic PAD, manifested by intermittent claudication, was diagnosed in 2 HIV-positive male patients (**LINK 1**). In both patients the ABI test confirmed PAD; an ultrasound scan revealed femoropopliteal occlusion. The patients differed regarding duration of infection and ARV therapy; at the time of the study they showed a good immune status and low viral load. Besides age and gender many cardiovascular risk factors were observed in both patients including heavy smoking, lipid disorders, a family history of cardiovascular diseases and in 1 patient, mild thrombocytosis and hyperhomocysteinaemia. In 1 patient there were indications for endarterectomy of the internal carotid artery (more than 80% stenosis); the other patient underwent femoral thromboendarterectomy. Both patients demonstrated higher than 0.9 mm cIMT values. The remaining patients (n = 109) were asymptomatic. There were no cases of low ABI values suggesting latent PAD.

Unusual symptoms, i.e. numbness, tingling, stabbing pain and muscle cramps in lower limbs, were observed in 28 patients. In all cases the angiologic assessment revealed no signs of peripheral arterial disease and normal ABI values. Among the asymptomatic patients no pulse within the dorsalis pedis artery (14) or posterior tibial artery (1) was observed; additional ultrasound scans of these arteries revealed normal patency and a typical triphasic flow spectrum.

**Figure 1:**
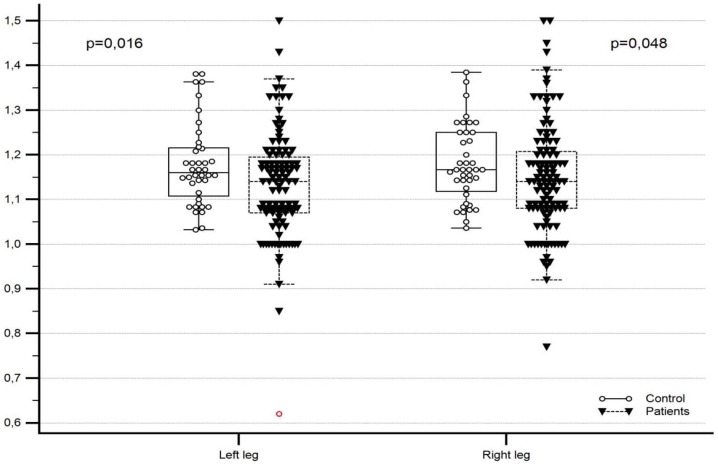
Comparison of ABI value as continuous variable between the study and control groups for the left and right lower limbs. Medians and IQR: Study group: Left leg 1.14 (1.07–1.20), right leg 1.14(1.08–1.21). Control group: Left leg 1.17 (1.12–1.25), right leg 1.16 (1.11–1.22).

The ABI median in HIV-positive group was significantly lower within the normal range and ABI values were more diversified, including abnormally high or low values compared to the HIV-negative subjects (Fig. 1). In the control group no cases of low ABI ≤1.0 were observed. In the study group 6 patients showed borderline ABI 0.91–0.99 and 5 patients showed ABI >1.4, which were not reported in any patient of the control group. The differences between the asymptomatic groups (HIV-positive and noninfected) regarding the sizes of specific subgroups according to the ABI categories were not significant (Table 3).

**Table 3:** Numbers of symptomatic and asymptomatic patients within the specific ABI categories regarding the study and control groups (chi-square test).

ABI category	Study group n(%)	Control group n(%)	P
Symptomatic low ABI ≤0.9	2 (1.8)	NA	NA
Asymptomatic low ABI ≤0.9	0 (0.0)	0 (0.0)	NS
Borderline ABI 0.91–0.99	6 (5.5)	0 (0.0)	NS
Normal ABI 1.00–1.30	88 (80.7)	34 (85.0)	NS
High ABI >1.3	15 (15.3)	6 (15.0)	NS
High ABI >1.4	5 (4.6)	0 (0.0)	NS
Altered ABI <1.0 and >1.3	23 (20.7)	6 (15.0)	NS

NA – not applicable

NS – non-significant

The statistical analyses focusing on the occurrence of cardiovascular risk factors as well as infection-related conditions in the subgroups showed that among HIV-positive patients with low ABI values (ABI <1), a family history of cardiovascular diseases is more frequently observed compared to patients with normal or high ABI values (p = 0.03). CRP concentration levels were higher in those patients, although this difference is not statistically significant (Table 4). In the ABI >1.3 category, metabolic syndrome tended to occur more frequently; this difference is not statistically significant (p = 0.083). No relationship was found between the ABI as a continuous variable and the evaluated risk factors **(LINK 2)**.

**Table 4:** Analysis of differences in cardiovascular risk factors and infection status between HIV-positive subgroups 3, 4 and 5 (Kruskall-Wallis test, chi-square test, Fisher's exact test).

Variable	Low ABI <1.0 n = 8	Normal ABI n = 88	High ABI >1.3 n = 15	P-value
Gender, M	5 (62.5)	55 (62.5)	11 (73.3)	0.69
Age, years	46 (34–52)	41.5 (34–46)	45 (35–49)	0.615
HIV infection time, years	7.5 (3.8–16.0)	9.5 (5.0–14.0)	8 (4.1–11.0)	0.61
ART treatment	7 (87.5)	77(87.5)	14 (93.3)	0.807
ART exposure time, years	6 (2–10)	6 (4–10)	7 (4–8)	0.907
CD4, cells/mm^3^	547 (441–708.5)	540 (421–735.5)	633 (426–982.3)	0.312
CD4 nadir, cells/mm^3^	264 (110–431)	230 (93–347)	148 (74–280)	0.63
HIV RNA, copies/ml	44.5 (40–2600)	50 (40–71)	40 (40–50)	0.504
HIV RNA zenith, copies/ml	118 (7–151)	37.7 (107–137)	89 (122–378)	0.787
Hypertension	5 (62.5)	35 (39.8)	7 (46.7)	0.43
BPs, mmHg	120 (105–135)	120 (110–140)	115 (105–130)	0.61
BPd, mmHg	75 (60–83)	80 (70–90)	80 (70–88)	0.488
Lipid disorders	7 (87.5)	59 (67.1)	11 (73.3)	0.456
TC, (mg/dl)	198.5 (164–216)	189(157.5–220)	193 (169–232)	0.822
LDL-C,(mg/dl)	128.5 (95–142.5)	101 (80–120)	115(78.3–132.3)	0.209
HDL-C, (mg/dl)	46 (37–51)	53 (42–64)	49 (39–50)	0.16
Non-HDL–C, (mg/dl)	155 (125–171)	128 (97–163)	150 (121–179)	0.239
TG, (mg/dl)	131 (96–193)	128 (88–188)	140 (107–182)	0.73
Lp(a), g/l	0.03 (0.02–0.09)	0.05 (0.03–0.18)	0.08 (0.02–0.36)	0.31
Family History	6 (75)	26 (29.5)	5 (33.3)	**0.03 (3) vs (4) (5)**
Smoking	8 (100)	66 (75)	12 (80)	0.437
Pack–years	33 (15–49)	15 (8–27)	17 (15–21)	0.23
CRP, mg/l	1.9 (0.6–12.0)	0.49 (0.18–1.23)	1.1 (0.3–3.3)	**0.08 (3) vs (4)**
Diabetes mellitus	0 (0)	3 (3.4)	0 (0)	0.9
Fasting glucose, mg/dl	89 (84–94)	91 (86–97)	88 (83–96)	0.727
Obesity	1 (12.5)	2 (2.3)	1 (6.7)	0.189
BMI	23.6 (22.3–25.6)	22.8 (21.0–24.9)	23.3 (20.5–26.4)	0.723
WHR	0.91 (0.825–1.03)	0.88 (0.83–0.95)	0.94 (0.91–0.96)	0.41
Metabolic syndrome IDF	2 (25)	14 (15.9)	6 (40)	**0.083 (4) vs (5)**
Homocystein, umol/l	12.1 (10.6–18.6)	12.8 (10.4–16.1)	12 (10.7–13.9)	0.65
Fibrinogen, g/l	2.9 (2.3–3.5)	2.6 (2.3–2.9)	2.5 (2.1–3.0)	0.515
D-dimer, ng/ml	326 (166–494)	218 (170–310)	170 (149–313)	0.37
Platelets, K/ul	242 (185–384)	214 (173–285)	223 (201–239)	0.618
cIMT mm	0.717 (0.633–0.899)	0.645 (0.587–0.785)	0.673 (0.586–0.762)	0.42
Cardiovascular risk ATPIII,%	5.5 (1.5–21.0)	2 (1.0–4.0)	3 (2.0–8.0)	0.255

Data shown as median and interquartile range or prevalence (number, percent).

A comparison of the subgroup with altered ABI and that with normal ABI showed that within this category higher LDL and non-HDL cholesterol levels, lower HDL cholesterol levels, higher CRP levels and more frequent cases of metabolic syndrome are observed, although these findings are not statistically significant (**LINK 3**).

Altered ABI values were significantly related to family history of cardiovascular disease, CRP, fibrinogen and cardiovascular risk assessed by the NCEP ATP III standard, but not to HIV infection (Table 5). In the study group, low HDL-cholesterol and metabolic syndrome occurrence are significantly related to altered ankle - brachial index (Table 6).

**Table 5:** Results of logistic regression analysis: association of risk factors with altered ankle-brachial index value in the summed study group and control group.

Variable	OR	95% CI	P-value
			
Gender M	1.07	0.46–2.51	0.87
Age	1.02	0.979–1.064	0.33
HIV infection	1.48	0.555–3.953	0.43
Hypertension	1.78	0.784–4.03	0.17
BPs	0.99	0.976–1.014	0.58
BPd	0.98	0.961–1.012	0.29
Lipid disorders	1.87	0.706–4.956	0.21
TC	1.00	0.992–1.0095	0.67
LDL-C	1.01	0.994–1.016	0.36
HDL-C	0.98	0.96–1.005	0.13
Non-HDL–C	1.00	0.995–1.013	0.37
TG	1.00	0.996–1.005	0.86
Lp(a)	0.37	0.041–3.396	0.38
Family History	2.61	1.12–6.05	**0.026**
Smoking	2.43	0.86–6.83	0.093
Pack–years	1.02	0.995–1.05	0.11
CRP	1.11	1.009–1.2195	**0.031**
Diabetes mellitus	NA	NA	NA
Glucose	0.97	0.933–1.012	0.16
Obesity	1.43	0.274–7.489	0.67
BMI	1.01	0.897–1.13	0.89
WHR	10.73	0.109–1057.02	0.31
Metabolic syndrome IDF	2.20	0.846–5.72	0.11
Homocystein	0.97	0.866–1.075	0.52
Fibrinogen	1.84	1.054–3.208	**0.032**
D-dimers	1.00	0.999–1.003	0.11
Platelets	1.00	0.997–1.007	0.39
cIMT	8.41	0.959–73.8	**0.055**
Cardiovascular risk NCEP ATP III	1.06	1.0005–1.129	**0.048**

**Table 6:** Results of logistic regression analysis: risk factors associated with altered ankle-brachial index value in the study group.

Variable	OR	95% CI	P-value
			
Gender M	1.07	0.46–2.51	0.87
Age	1.02	0.979–1.064	0.33
HIV infection	1.48	0.555–3.953	0.43
Hypertension	1.78	0.784–4.03	0.17
BPs	0.99	0.976–1.014	0.58
BPd	0.98	0.961–1.012	0.29
Lipid disorders	1.87	0.706–4.956	0.21
TC	1.00	0.992–1.0095	0.67
LDL-C	1.01	0.994–1.016	0.36
HDL-C	0.98	0.96–1.005	0.13
Non-HDL–C	1.00	0.995–1.013	0.37
TG	1.00	0.996–1.005	0.86
Lp(a)	0.37	0.041–3.396	0.38
Family History	2.61	1.12–6.05	**0.026**
Smoking	2.43	0.86–6.83	0.093
Pack–years	1.02	0.995–1.05	0.11
CRP	1.11	1.009–1.2195	**0.031**
Diabetes mellitus	NA	NA	NA
Glucose	0.97	0.933–1.012	0.16
Obesity	1.43	0.274–7.489	0.67
BMI	1.01	0.897–1.13	0.89
WHR	10.73	0.109–1057.02	0.31
Metabolic syndrome IDF	2.20	0.846–5.72	0.11
Homocystein	0.97	0.866–1.075	0.52
Fibrinogen	1.84	1.054–3.208	**0.032**
D-dimers	1.00	0.999–1.003	0.11
Platelets	1.00	0.997–1.007	0.39
cIMT	8.41	0.959–73.8	**0.055**
Cardiovascular risk NCEP ATP III	1.06	1.0005–1.129	**0.048**

## Discussion

The prevalence rate of PAD in our study group was low; however, nearly one in five asymptomatic, young or middle-aged HIV-patients showed various ABI alterations (19.3% of asymptomatic subjects). We did not find any relation between the ABI alterations and the status or course of HIV infection nor the duration of antiretroviral treatment. Previous studies have shown that peripheral arterial disease diagnosed on the basis of the resting ABI measurement occurs more frequently in HIV-positive patients than in the general population [12], [13], [14], [15]. Those studies differed regarding patients' age, race and gender, the inclusion criteria and the ABI testing method. In most of them the authors reported that asymptomatic or symptomatic PAD occurred more frequently in HIV-positive patients than in the overall population. There are also contradictory observations suggesting that there are no significant differences in this respect between the general population and the cohorts of HIV-positive patients, and there is no need for additional diagnostic procedures other than the approved guidelines for the overall population [16], [17].

In the studied group with many cardiovascular risk factors the most typical were smoking, lipid disorders, followed by family history of cardiovascular diseases and hypertension. The prevalence of risk factors in the adult Polish population shows a different pattern, with lipid disorders being the strongest, followed by arterial hypertension and eventually by smoking [18].

A current valid and widely used PAD diagnostic method is the resting ABI test [6]. Resting ABI ≤0.9 is considered as an indicator of hemodynamically significant arterial stenosis. The test is non-invasive, simple, reproducible, inexpensive and available in every medical facility.

In the presented cohort, young and middle-aged adults were included, with a considerably higher number of male patients. This is the first study in Poland focused on PAD occurrence in HIV-positive patients. The prevalence rate of symptomatic PAD at the stage of intermittent claudication confirmed by the ABI test was 1.8% and only referred to two middle-aged (52 on average) men with intermittent claudication and many cardiovascular risk factors. In both patients, atherosclerotic lesions in other vascular areas and high cIMT values were observed.

The prevalence of symptomatic PAD in the studied group of HIV-positive people was the lowest when compared to previous studies, where it was assessed as 2.7, 5.1 and 8.7% [14], [15], [16]. However, the patients of the above cohorts were older by 11, 17 and 8 years on average, respectively. Data on the PAD prevalence and ABI value abnormalities among the Polish general population are limited. The only available study on epidemiology of PAD in Poland refers to on overall population over 20 years of age. The findings indicate the age-related occurance of the symptomatic disease with the prevalence rate of 2% [19].

According to TASC II the prevalence rates of intermittent claudication would appear to increase from about 3% in patients aged 40 to 6% in patients aged 60 years in the general population [6]. The prevalence of symptomatic PAD in the presented cohort is comparable to that demonstrated in the study of patients within the same age range in a non-infected general population [20], [21] and lower than the prevalence reported by the authors of studies involving HIV-positive cohorts [14], [15], [16]. A study conducted with the same method in the overall population of middle-aged subjects showed similar PAD prevalence rates for Caucasians: 2.3% for men and 3.2% for women. Compared to the present study, the average age was higher by approximately 15 years, which may explain different PAD prevalence rates for both genders and for women in particular [22]. In the National Health and Nutrition Examination Survey, the PAD prevalence rate in patients aged 40–49 and 50–55 was 0.6–1.1% and 1.9–3.1%, respectively [23]. Due to the small number of diagnosed PAD cases in the study group, a comparison of the findings and the epidemiological data concerning the general population cannot be performed – the prevalence rates seem to be comparable.

Among HIV-positive patients with ABI <1.0, there were more cases with a family history compared to patients with ABI >1.0. The study conducted by Periard et al. showed a relation between the family history and PAD in HIV-positive patients in addition to other conventional risk factors (age, smoking, lipid disorders, diabetes, hypertension, metabolic syndrome) and low CD4 cell counts [14]. The results of our study are consistent with the observations of Valentine et al., who in a study of twins demonstrated that in the general population of young adults a very strong risk factor which determines premature PAD development is the family history. Although smoking was a dominant risk factor, premature atherosclerotic lesions were also observed in asymptomatic non-smokers with a family history of the disease [24].

In the overall population, asymptomatic PAD prevalence is 3 to 4 times higher than that of symptomatic PAD [6], [25]. In the Framingham Offspring Study, low ABI values were reported in 3.6% of a non-selected study group aged 59 on average [25]. In the present study of 109 infected patients and the control group, no cases of asymptomatic PAD were observed. No observation of asymptomatic PAD in our cohort is the lowest finding as compared to the results presented so far for HIV-positive populations. The prevalence rates of asymptomatic peripheral arterial disease vary from 1.1% to 6,3% for middle-aged cohorts [14], [26]. In the oldest studied group of HIV-positive patients (average age 58.6 years) diagnosed with PAD the prevalence rate of latent peripheral arterial disease with ABI ≤0.9 was 5.1% [15]. Regarding age and gender, our study group is very similar to the group of Spanish HIV-infected patients, in which asymptomatic PAD was diagnosed in 6,3% of the cohort. This large difference between the two studies may be explained by a slightly different age distribution, two times more frequent occurrence of diabetes and almost four times more frequent occurrence of obesity [26].

Based on post-exercise ABI measurements, Qaqa et al. diagnosed PAD in 26.5% of HIV-positive patients in an ethnically different and older patient group than ours [27]. Periard et al., also using post-exercise ABI measurements and slightly different diagnostic criteria, diagnosed PAD in 10.9% of the patients [14]. According to the guidelines for the general population the post-exercise ABI test is recommended when a normal resting ABI value is accompanied by symptoms suggesting intermittent claudication [6]. The results of both studies with post-exercise ABI measurements demonstrate a need for better understanding of prognostic significance as well as application of this method for diagnostic purposes in the case of HIV-positive populations [14], [27].

The median value of ABI in HIV-positive patients was significantly lower (though within the normal range) and more varied compared to the HIV-negative subjects. Borderline ABI values were observed in 5.5% of the study subgroup of asymptomatic HIV-positive patients (in the whole group – 5.4%). In the study conducted by Jang et al., this proportion was higher: 18% in a cohort of HIV-positive patients older by 7 years on average [13]. Currently, the borderline ABI value is considered prognostically significant and is associated with 10% higher cardiovascular mortality [4].

Altered high ABI >1.3 (above 1.4 in particular) is considered as a sign of rigidity and noncompressibility of lower limb artery walls. It is mostly observed in diabetic patients with media calcification as well as in patients with metabolic syndrome, and it is associated with conventional risk factors. Similarly to the low values, diagnostic and prognostic significance is also attributed to high ABI values [28]. In the study group, ABI >1.3 was observed as often as in the control group, while ABI >1.4 was only observed in the HIV-positive group. In a study on PAD in HIV-positive patients, Jang et al. obtained the same results: 15% of the subjects showed ABI >1.3 and, comparably to the present cohort, twice as many high ABI values were observed in men [13]. Additionally, in this subgroup a tendency for more frequent cases of metabolic syndrome and higher CRP levels was seen compared to the group with normal ABI values, but the differences were not statistically significant. The study conducted by Tomiyama et al. based on pulse wave velocity testing showed that metabolic syndrome is related to arterial stiffness and noncompressibility, while an increased hsCRP level is a significant, positive predictive factor for arterial stiffness [29].

Very high ABI >1.4 values were reported in 4.6% of the study group. According to other authors, ABI >1.4 prevalence rates were 0.5 to 7.1% in the HIV-positive groups, which was affected by smoking as well as by being under- and overweight [30]. According to Gutierrez, patients with at least two risk factors and ABI >1.4 showed lower LDL cholesterol levels and a lower risk assessed with the Framingham scale compared to patients with normal ABI values [31]. We also observed low cardiovascular risk, the lowest levels of all cholesterol fractions and triglycerides in the subgroup with ABI >1.4. Due to the small sample size, these phenomena were not statistically analyzed.

In addition to the above observations, patients with altered (low or high) ABI values more frequently showed signs of metabolic syndrome, higher CRP, LDL-C, non-HDL-C levels and lower HDL-C levels compared to patients with normal ABI values and show a strong relation to low HDL-C level and metabolic syndrome. These findings are consistent with the results obtained by Jang et al. with regard to CRP levels [13]. Biochemical disorders (components of the metabolic syndrome) are associated with a chronic, subclinical inflammatory process and significantly higher cardiovascular risk [29].

In the present study we did not find any relation between the ABI alterations and the status of HIV infection or of antiretroviral treatment. The results are consistent with Sharma's reports that precluded an impact of the infection status on ABI values [30]. However, other authors reported relation between ABI values and current low CD4 cell counts, advanced HIV infection or protease inhibitor treatment [12], [14], [26], [31].

The prevalence rate of PAD in our study group was extremely low; however, nearly one in five asymptomatic, young or middle-aged HIV-patients showed various ABI alterations (19.3% of asymptomatic subjects). Our findings and other authors' reports indicate the need for PAD screening in HIV-positive patients.The resting ABI measurement, as in the general population is the method of choice [6]. In the case of altered ABI values, evaluation of cardiovascular risk and implementation of further diagnostic, preventive or therapeutic procedures are necessary.

This study has some limitations. The most important limitation was the relatively small study group although it represented 14.8% of the patients attending the Outpatient Acquired Immune Disorders Clinic and in terms of the characteristics of HIV infection was representative of the entire HIV-positive population in Poland. After dividing the group into subgroups depending on the value of ABI, we had to omit the smallest subgroups in the statistical analysis. Due to the small size of the control group, we could not achieve adequate power of our statistical analysis, and many parameters were on the border of statistical significance. There was no possibility for comparison of our results with another contemporary analysis in the Polish general population, with only data from the 1980s available. Accordingly, in the discussion of our results, we compared them with the results of the most important worldwide research concerning the prevalence of PAD in the general population.

## Conclusions

In the group of young and middle-aged Polish HIV-positive patients, symptomatic PAD prevalence is comparable with that observed in the overall population and is lower than in the other HIV-positive studies as well. Among asymptomatic patients, PAD is not observed. The ABI value is significantly lower and more varied compared to the HIV-negative subjects. Compared to the gender- and age-matched asymptomatic noninfected group, altered borderline or high ABI >1.4 values tend to be more prevalent in the asymptomatic HIV-positive subjects. Low ABI <1.0 values are strongly related to a family history of cardiovascular diseases. Abnormalities of ankle-brachial index are associated with low high-density lipoprotein cholesterol level and metabolic syndrome. The relationship between observed ABI alterations and the status of HIV infection or antiretroviral treatment needs further studies.
